# An analysis of substantiated complaints made about incidents of poor livestock welfare, in Victoria, Australia.

**DOI:** 10.3389/fvets.2023.1242134

**Published:** 2023-08-29

**Authors:** Natarsha Williams, Sarah Chaplin, Lauren Hemsworth, Richard Shephard, Andrew Fisher

**Affiliations:** ^1^Animal Welfare Science Centre, Melbourne Veterinary School, Faculty of Science, University of Melbourne, Parkville, VIC, Australia; ^2^Department of Energy, Environment and Climate Action, Tatura, VIC, Australia; ^3^Herd Health Pty Ltd., Maffra, VIC, Australia

**Keywords:** livestock, animal welfare, rainfall, extensive farming, animal welfare complaints

## Abstract

Incidents of poor welfare on farm in Victoria, Australia, are generally identified during an investigation that follows receipt of a complaint. Using deidentified records of complaints received by the Victoria State Government between 2011 and 2020, this study aimed to describe the source, number and the relationship between rainfall/stock prices and substantiated welfare complaints (SWC). Only incidents involving non-dairy cattle, sheep and goats in extensive farming systems will be considered. The main source of complaints received by the Victorian Government is the general public. Almost half of all complaints were made for cattle (48%), 39% for sheep, 11% for mixed species, and 2% for goats. The number of SWC varied between months, each year and across the different regions of Victoria. The ratio of the actual mean rainfall of the last three seasons to the long-term mean of the last three seasons of rainfall (RL3SR) and livestock prices together were the best predictors of the total number of SWC (adjusted R square value for heavy lamb-RL3SR was highest (0.590), followed by merino lamb-RL3SR (0.588), goat-RL3SR (0.545) and steer-RL3SR (0.478) all were significant (*p ≤ 0.05*)). The rainfall by region and town were not good predictors of the number of SWC. There was a correlation between rainfall and the number of SWC, possibly due to changes in pasture availability. Favorable seasonal conditions however, were not protective of livestock welfare and it is likely a number of factors may be implicated.

## 1. Introduction

Incidents of poor livestock welfare continue to occur in extensive faming systems (1–5). The issues mostly involve a chronic failure to provide basic care, including food, water, treatment and a suitable environment (2, 5–7). By contrast, incidences of malicious abuse are rare (4, 5). Both small and large farms may be affected, impacting on individuals to hundreds of animals (2, 5, 8).

In Australia, the State and Territory Governments and the Royal Society for the Prevention of Cruelty to Animals (RSPCA) predominantly enforce animal welfare standards through administration of the relevant legislation (9). In contrast, other countries regulate animal welfare at a national level with or without the assistance from local government and prevention of cruelty to animals agencies, for example New Zealand (10), Ireland (11), America (12) and United Kingdom (13). The responding agency may vary depending on the species and number of animals affected. Each jurisdiction in Australia has its own animal welfare legislation (9). In Victoria, this legislation is the Prevention of Cruelty to Animals Act 1986 (POCTAA) (14), however a new animal welfare act is being developed (15). Countries in the European Union (EU) perform animal welfare inspections as part of their requirements as member states of the EU, to assess compliance with the European standards for animal welfare (2, 3, 16).

In Victoria, there were approximately 10,000 beef cattle businesses, farming 2 million beef cattle that produced 495,000 tonnes of beef and veal in 2019–20 (17). The majority of cattle are raised in extensive pasture-based systems in Victoria, and are set stocked with rotational grazing. Cows are generally bred for set periods and calve in autumn or spring. The cattle are mostly *Bos taurus* including many British breeds (18). There were 8,600 sheep businesses in Victoria, with 15 million sheep that produced 314,000 tonnes of carcass weight sheep meat in 2019–20 (19), and 92,000 tonnes of wool in 2020–21 (20). The vast majority of sheep are raised on pasture and are joined to lamb seasonally in either spring or autumn. The breed used depends on the production system of wool, meat or both. Goat farming is much smaller in Victoria with only 38 self declared goat farmers in the 2016 census (21). However, Victoria is the largest processor of goat meat, with ≥96% of the goats coming into Victoria from other jurisdctions (22) as cited by (23). There are also a small number of goats kept for their fiber (24, 25).

Animal welfare investigations in Victoria occur in response to receiving a complaint (26, 27). Animal welfare complaints can be made to Agriculture Victoria by telephone and / or email to the RSPCA via an online form or telephone (28). An initial visit determines if there has been a breach in the legislation, and if so, the complaint is substantiated. The investigative process, including the use of legal instruments, collection of evidence and the recording of all relevant events has been described in another study (29). Records made as a result of animal welfare complaints and inspections, are likely to be a valuable resource to improving the understanding of farm animal welfare issues. Firstly, the nature and extent of the issues can be better understood, and the key challenges identified. Secondly, potential links between certain farmer and farm characteristics and the incidence of poor welfare may be identified (30).

Neglect of animals on farm has been found to be associated with farm production related issues, such as failing to: provide adequate treatment (3, 5, 16), nutrition (3, 5), supervision (2, 3, 16) or grouping animals according to their age and gender and uncontrolled breeding (31). Family and socio economic based factors have also been identified including: age (32), family loss (33), financial issues (32, 34, 35), stress (32, 34), physical and social isolation (36), reluctance to ask for help (32), relationship issues (32, 34), the farmer’s physical (33–35) and mental health (32–35). Neglect of livestock may not be intentional (33) but instead may be ‘a multi-causal phenomenon’ where problems tend to accumulate over time (34). Underlying problems need to be identified and addressed for intervention to be successful at improving poor welfare (30), p. 18. In addition, through improved understanding of the problems associated with reoffending, more targeted intervention could be employed to manage these high risk situations and ensure effective monitoring programs are developed (30).

Extensive farming systems are often considered to provide livestock with better welfare (7, 37–39), with the opportunity to express natural behaviors, while also benefiting from better health and less stress (38). However, this can be accompanied by vulnerability to the unpredictability of the environment (40), including variability in the quality and quantity of pasture (7). Australia’s climate varies considerably between regions and over time (41), and droughts are likely to occur more often with climate change (42), further increasing the risk of times when pasture is limiting. Livestock and feed prices also fluctuate between years (43), impacting the financial challenge of supplementary feeding to make up for pasture shortfalls. Other welfare challenges in extensive systems include water scarcity, high temperatures (7) and the risk of predators (44, 45).

Through identifying the circumstances around which poor livestock welfare incidents occur, improved strategies of intervention, extension and prevention may be implemented. Potentially, there may be factors or challenges that occur more commonly on properties where the welfare of the livestock is poor and these may be considered as risks, and used to predict situations where negative welfare outcomes are likely. This approach has been used to predict human inpatient aggression (46), high and low risk offenders and reoffenders of domestic violence (47), and risk and safety assessments in child welfare (48).

The aim of this study was to better understand the main source and timing of substantiated welfare complaints (SWC) affecting non-dairy cattle, sheep or goats in extensive farming systems, in Victoria, Australia. This study also aimed to explore the relationship between rainfall, stock prices and SWC including rainfall across Victoria, its regions and towns. It was hypothesized that the number of SWC would be correlated to rainfall, because of the direct link between rain, pasture growth, and subsequent feed availability to meet livestock requirements.

## 2. Methodology

**Figure 1:**
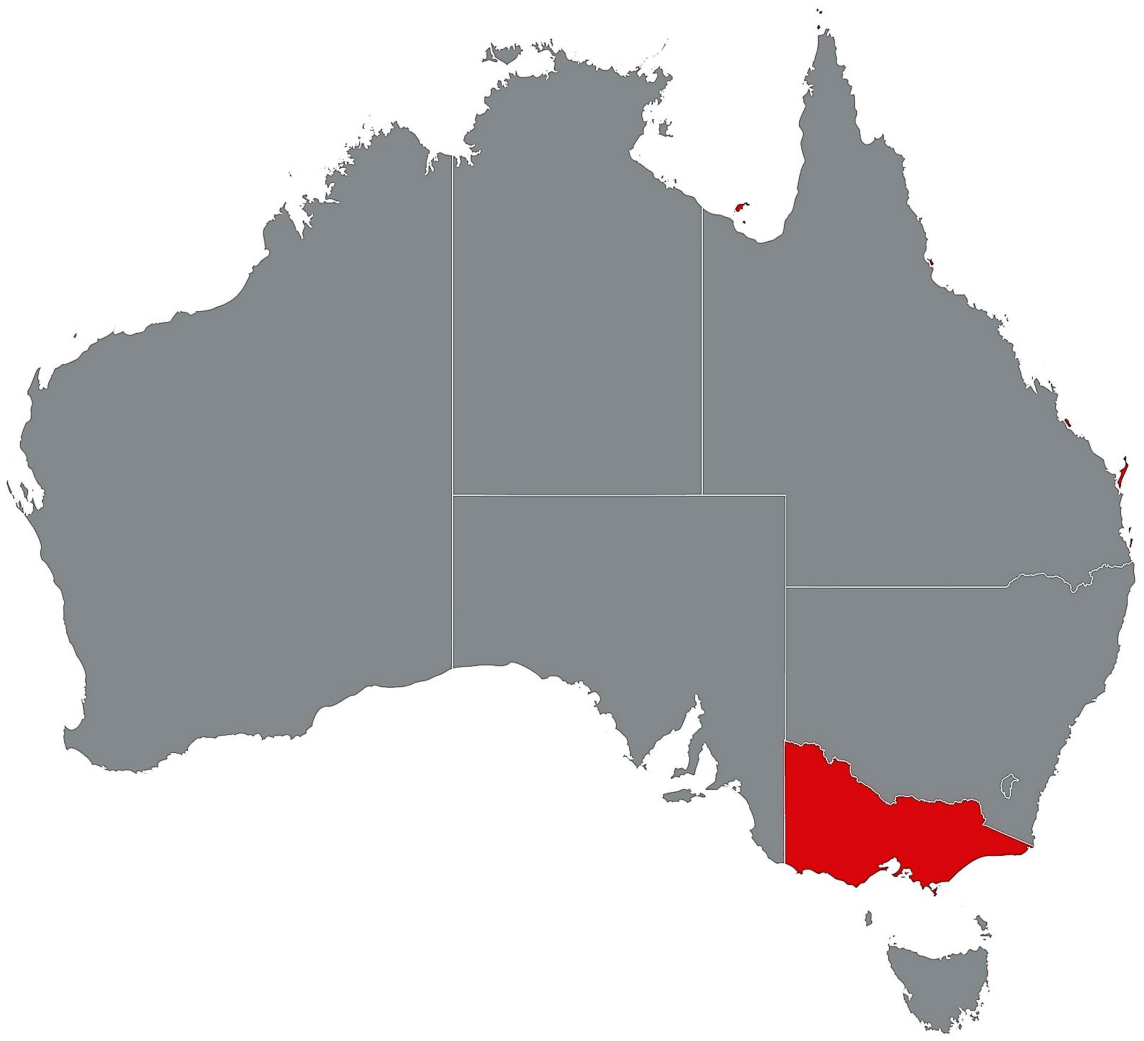
Map of Australia, with Victoria highlighted in red.

Human ethics approval was not required for this study, as there was no direct engagement with farmers and the data was historical and deidentified. The livestock in the dataset analyzed in this study comprised non-dairy cattle, sheep and goats. This study is focused on data from the state of Victoria in the south-east of Australia, highlighted as red in Figure 1.

Animal welfare complaints received by Agriculture Victoria are recorded electronically in an Animal Welfare Log (AWL), a system that was established in 2009. Under a Memorandum of Understanding (MoU) with RSPCA Victoria, Agriculture Victoria investigates complaints about livestock where there are more than 10 animals and RSPCA Victoria investigates complaints about small number of livestock, horses and domestic animals (49). If RSPCA Victoria receives complaints about livestock where there are more than 10 animals, under the MoU, they will refer the complaint to Agriculture Victoria, and vice versa.

This study focused on non-dairy cattle, sheep and goats in extensive farming systems because of the similarities in the production systems, compared to dairy farms and intensive farms that are quite different. One of the aims of this study was to identify risk factors that might be used to predict livestock at risk of poor welfare. It was determined that a narrower scope of species type and production system would increase the likelihood that the factors would be better predictors.

### 2.1. Data preparation

Animal Welfare Log data was provided in two data sets by Agriculture Victoria, one containing data from 2009 to 2019 and one for 2020. The two data sets were combined, resulting in a total of 9,512 deidentified complaints from 2009 to 2020. The name of the town where the alleged incident occurred was retained. There were only 13 records from 2009 to 2010. These were not included in analysis as they were likely only a small fraction of the incidents in that timeframe. The final data period analyzed was from 1st January 2011 to 31st December 2020.

### 2.2. Data analysis-general

Initially, using all complaint records from all species from 2011 to 2020, complaints were divided into those that were substantiated (i.e., a breach in the legislation) and other, using the following criteria.

1. Substantiated - substantiated, advisory letter, notice to comply, warning letter, legal brief for prosecution/infringement notice.
2. Other - unsubstantiated, no further action, surveillance for complaints not substantiated on first visit, managed with phone call, extension material, no visits, referred to another agency (RSPCA), insufficient information to action complaint or determine the outcome of the investigation.

‘Substantiated complaints for key livestock, in extensive farming systems’ (SWC) were retained for further analysis. The species categories used were cattle, sheep, goats and mixed (where there was more than one species present and at least one was a key livestock species). Complaints for all other species and dairy breeds of key livestock were removed. It is possible that some were missed as the separate ‘dairy cattle’ and ‘beef cattle’ categories were not used until early 2016. Prior to that and continuing until July 2017 a combined ‘cattle’ category was used. Complaints about poor welfare in intensive farming systems, abattoirs, sale yards and knackeries were removed from further analysis as they were not in the scope of this project. Complaints received for cases already under investigation were identified as an ‘existing allegation’ and then removed to avoid duplicate reporting of the same incident. As this categorization was not extensively used until 2017, repeat complaints prior to then could only be identified if the allegation comments referred to existing welfare concerns. Finally, the free text in the allegation comment box was reviewed for each remaining complaint, and further entries that did not meet the selection criteria were identified and removed. This included complaints about non-target animals, incidents already under investigation, complaints involving a single animal, incidents in farming systems that were not extensive or entries that were repeat complaints for existing investigations.

Using just the SWC (*n* = 2,486), the overall proportion of complaints for cattle, sheep, goats and mixed species were calculated. The proportion of complaints for each species was then compared by month and by year. The number of complaints received from each source was determined. Ten different source options used over the 10 years of records were merged to form five categories as detailed below:

- Government (Rangers, National, State and Local government)
- Member of the public
- RSPCA (Vic)
- Welfare activist groups
- Other - veterinarians, police, self-observed.

**Figure 2:**
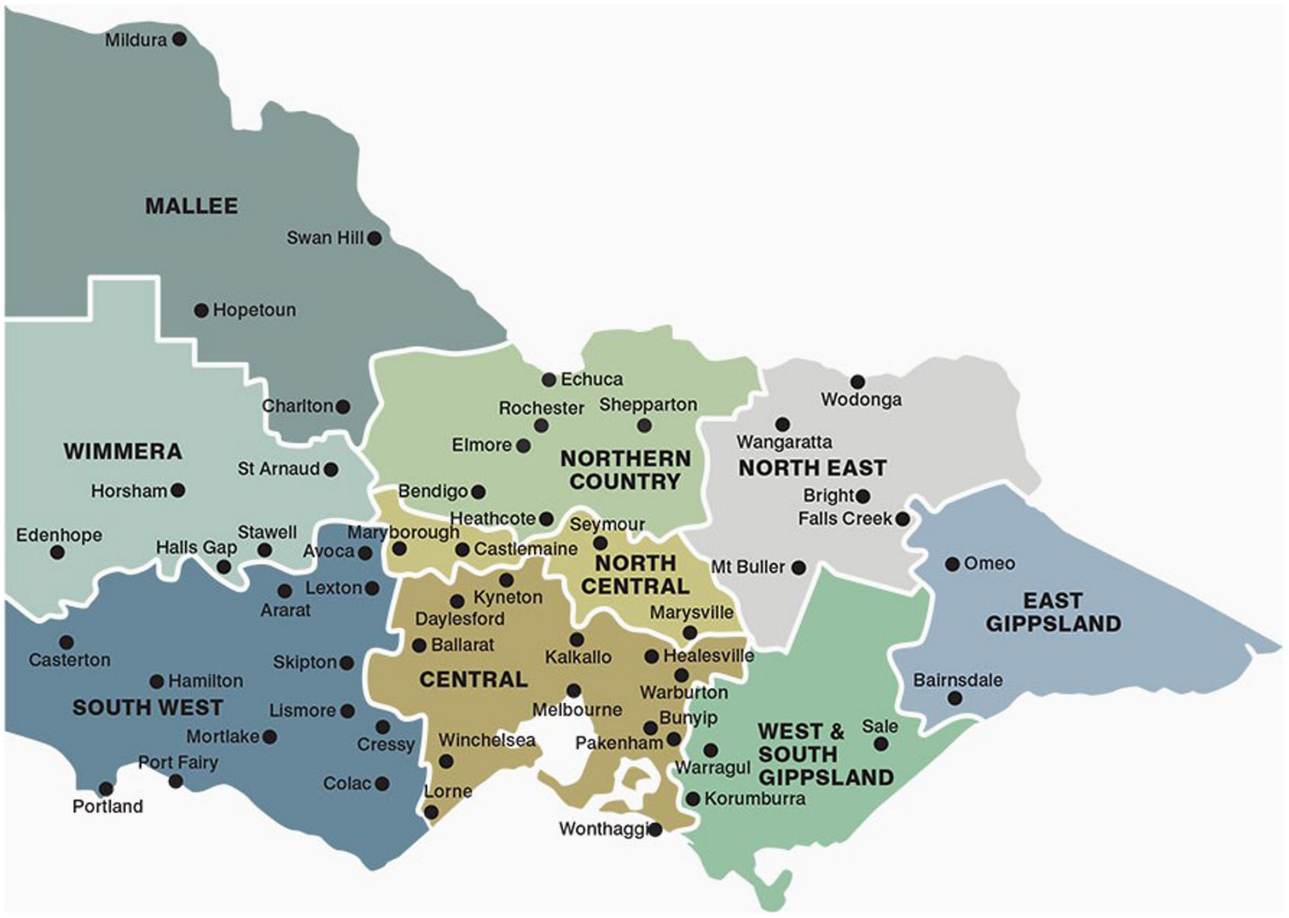
Regions of Victoria (50). Reproduced with permission from the CFA.

The number of complaints for each species category was calculated. In Victoria, animal welfare complaints are allocated to the nearest government office for investigation. Twenty-six different offices were open at some point over the data collection period. For analysis purposes, the offices were grouped into the Country Fire Authority (CFA) regions of Victoria (Figure 2) to mitigate the inconsistencies in the data as the number of offices and staff varied across the data collection period. The proportion of complaints received in each region were calculated and compared overall, per year using Microsoft® Excel® for Office 365.

### 2.3. Data analysis–association between SWC and rainfall

To determine if there was a significant association between rainfall and SWC, the impact of the rain was considered annually, seasonally and across the state, by region and town. Ratios between the actual rain that fell in the selected period to the mean rainfall for the same period over several years were used to describe variations in rainfall. In addition, seasonally adjusted regression allowed for the typical variation in rainfall that occurs between seasons every year normally. The relationship between the number of SWC and rainfall data was examined using rainfall data downloaded from the Bureau of Meteorology (BOM) (51), using the regression function on Microsoft® Excel® for Office 365. A summary of the acronyms is included in Table 1.

**Table 1:** Summary of acronyms used in analysis of rainfall and SWC.

Acronym	Definition
AR	Annual rainfall
LYAR	Last year’s annual rainfall
L2YAR	Last two year’s annual rainfall
SR	Seasonal rainfall
LSR	Last season’s rainfall
L2SR	Last two season’s rainfall
L3SR	Last three season’s rainfall
RL3SR	Ratio L3SR and m. L3SR
m.	Mean

Using Victorian rainfall data, the relationship between the number of SWC per year and the total annual rainfall (AR) on that year and the total of the previous year (LYAR), the mean of that year’s AR and the previous year’s annual rainfall (LYAR) and the mean of the L2YAR were examined. Lastly, the relationship between the number of SWC per season to the ratio of the actual rainfall in that same season to the mean rainfall for: the season (SR), the last season (LSR), the mean of the last two seasons rainfall (L2SR) and the mean of the last three seasons of rainfall (L3SR), in Victoria, were examined using seasonal adjustment regression. The Victorian mean SR was available on the BOM website and had been calculated based on rainfall data from 1961 to 1990 (51).

The association between local rainfall and SWC was assessed for two government offices: Ballarat and Tatura. They were selected as the historical rainfall data were relatively complete and both offices had a high proportion of the state’s SWC with 15.8% in Ballarat (the highest of any office) and Tatura 6.2% (fourth of all the offices). In addition, the mean AR was quite different in both towns with 689 mm in Ballarat (52) and 483 mm in Tatura (53), and they were approximately 220 kilometers apart, providing two quite different towns to compare. The seasonal rainfall for the towns of Ballarat (52) and Tatura (53) were calculated by totaling the corresponding monthly rainfall from the BOM website, according to the Southern Hemisphere seasons (summer – December, January and February). The mean SR for Ballarat was calculated using the historical monthly rainfall means from the BOM website based on data from 1908 to 2022. The mean SR for Tatura was based on the mean monthly rainfall for 2011 to 2020. Seasonally adjusted regression was used to analyze the relationship between the SWC for a season compared to the ratio of the actual to mean SR, LSR, mean of L2SR and L3SR for both Tatura and Ballarat.

The number of SWC for each region per year were compared to the ratio of the actual AR to the mean AR, the last year (LYAR) and the mean AR and LYAR. The north central region was excluded as there was only data from 2011 to 2016. As mean regional rainfall data was not readily accessible, the mean AR of two weather stations within each region was used as the AR rainfall. Weather stations were selected based on the completeness of the AR data and the distance between the stations, with a preference for locations relatively far apart. The weather stations and the number of years of data used to calculate the mean AR for each region can be seen in the Supplementary material.

The monthly mean key livestock prices, in cents per kilogram carcass weight, were obtained from Meat and Livestock Australia (43). These included saleyard sheep and lamb indicators for heavy lambs (22 + kg) and merino lambs (16-22 kg), saleyard cattle indicators for medium steers (4-500 kg) and over the hooks prices for goats (20.1 + kg) (43). The mean of the monthly values was calculated to create mean seasonal prices based on the Southern Hemisphere seasons. Using seasonally adjusted regression, the relationship between the number of SWC for Victoria to the mean seasonal price per kilogram of key livestock and to the ratio of the L3SR and m. L3SR and the seasonal stock price to the SWC for the same season were investigated.

## 3. Results

### 3.1. General analysis

**Figure 3:**
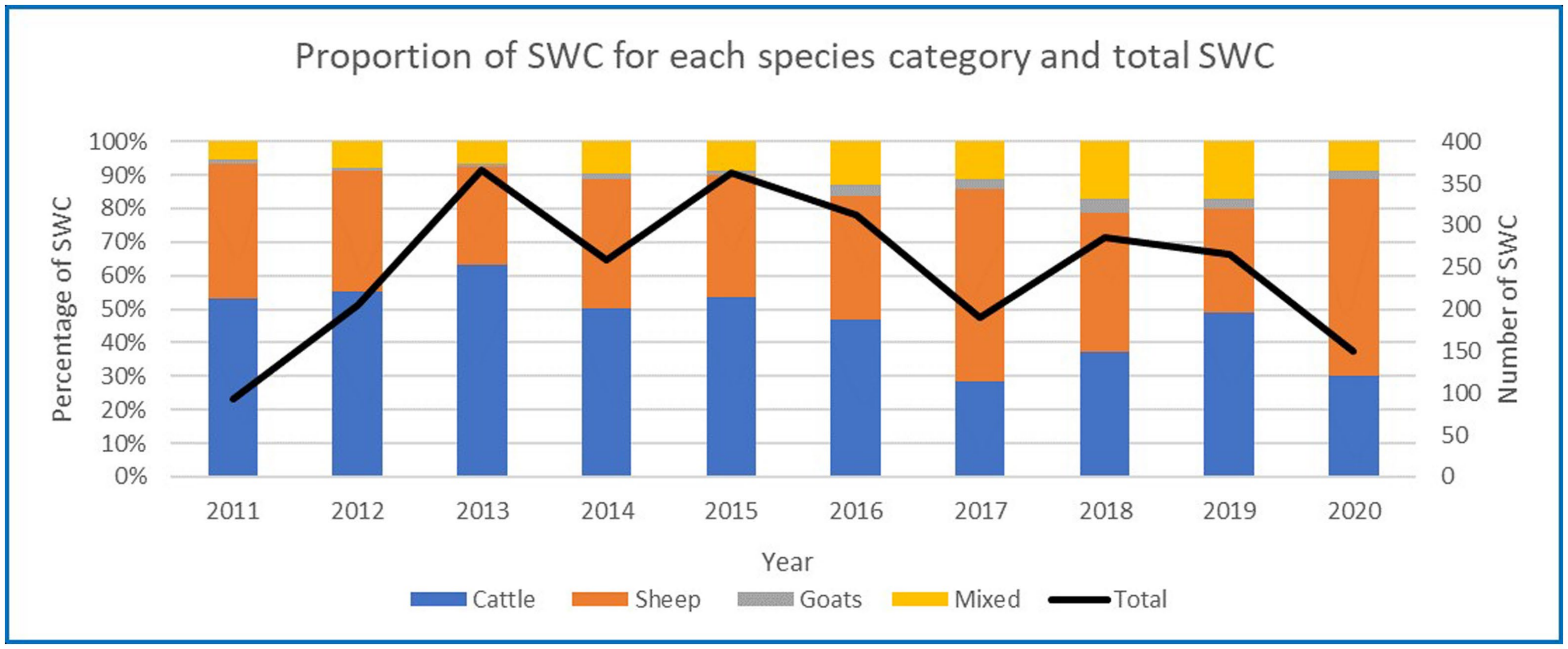
Proportion of SWC for each key species and total number of substantiated complaints.

Most substantiated welfare complaints came from the RSPCA (45%), followed by members of the public (35%), government (14%), other (4%) and welfare activist groups (1%). The highest number of SWC in a year were received in 2013 (366 SWC) and 2015 (363 SWC) (Figure 3). The lowest number of SWC in a year was recorded in the first and last years of the data collection (2011 = 92 SWC, 2020 = 149 SWC).

Almost half of all SWC received about key livestock were for cattle (48%), followed by sheep (39%), mixed species (11%) and goats (2%). The proportion of SWC for each key livestock species fluctuated over the 10 years (Figure 3) by up to 35%.

**Figure 4:**
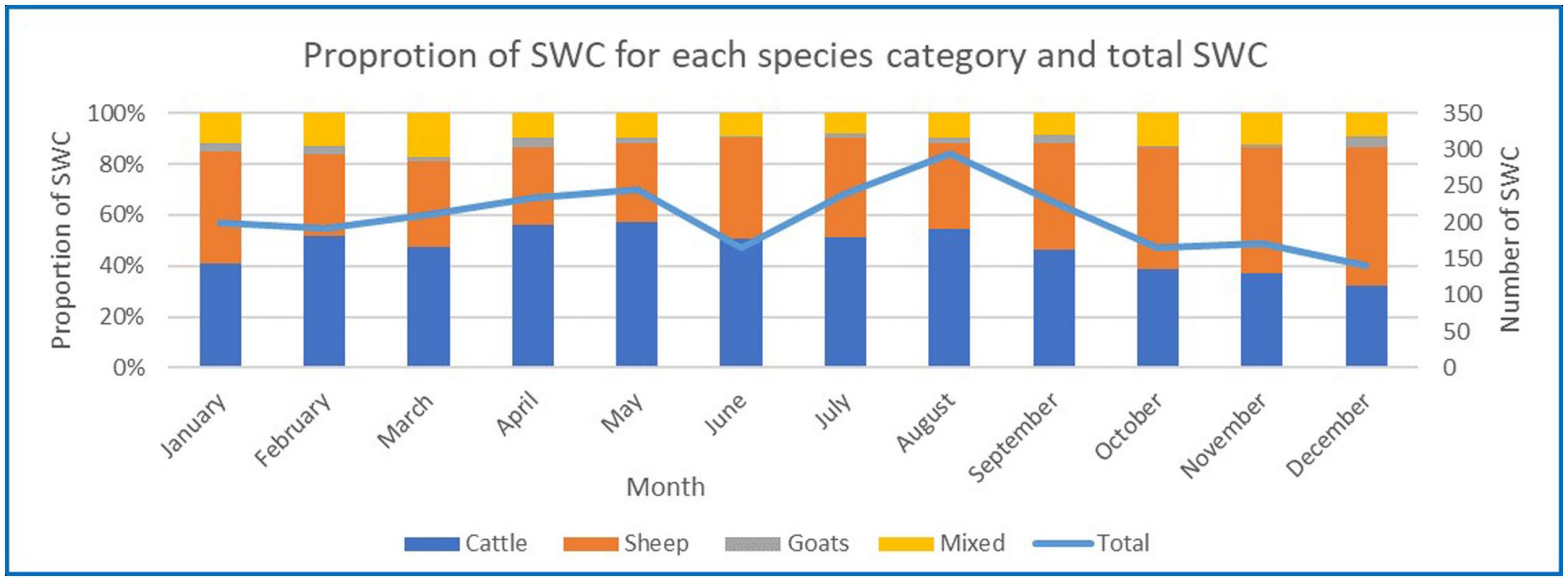
Number of SWC for each key species based on the months of the year.

The lowest number of SWC was in December then June and the highest number in August, as seen in Figure 4. The proportion of SWC for each species category varied between the months. The proportion of SWC for cattle varied from 57 to 32%, for sheep 54–31%, mixed 17–8% and goats 4–1% over the 12-month period.

Almost one third of SWC from 2011 to 2020 were in the Central region (31%), followed by Northern Country (21%), South West (16%), West South Gippsland (13%) and the North East (9%) regions of Victoria. Ten percent of SWC were in the remaining five regions combined. The proportion of SWC in the regions varied by less than 10 to 24% over the 10 years. The variation in SWC, by region, over time can be seen in the Supplementary material Figures 1 and 2.

### 3.2. Association between SWC and rainfall and stock price

Using seasonally adjusted regression, the ratio between the actual and mean rainfall in the last two seasons and three seasons, for Victoria were the best predictors of the number of SWC with an adjusted R square of 0.375 and 0.401, respectively (*p* ≤ 0.05) Table 2. The regression coefficients were all negative, indicating as rainfall decreased the number of SWC increased. The variation in winter rainfall was the greatest predictor of the number of SWC, excluding the seasonal rainfall (SR) regression which was not significant. There was a small but significant correlation with summer and SWC for the ratio of the L2SR and the m. L2SR.

**Table 2:** Results of regression analysis comparing annual and seasonal rainfall to the number of SWC.

Variable compared to SWC	Adjusted R Square	Significance F	Standard Error	Regression coefficient (*p* value)			
Victoria’s annual rainfall							
Ratio of AR to m. AR	0.139	0.156	83.257	−255.306 (*p* > 0.05)			
Ratio of LYAR and m. AR	0.169	0.131	81.813	−222.776 (*p* > 0.05)			
Ratio m. AR & LYAR to m. AR	0.301	0.058	75.023	−362.001 (*p* > 0.05)			
Ratio of m. L2YAR and m. AR	−0.11	0.820	94.855	−48.284 (*p* > 0.05)			
Victorian seasonal rainfall				Winter	Spring	Summer	Autumn
Ratio of SR to m. SR	−0.006	0.453	26.87	−26.471 (*p* > 0.05)	−16.820 (*p* > 0.05)	−12.466 (*p* > 0.05)	−1.233 (*p* > 0.05)
Ratio of LYSR to m. LYSR	**0.176**	0.031	24.31	**−34.687 (*p* ≤ 0.05)**	−13.395 (*p* > 0.05)	−17.032 (*p* > 0.05)	5.957 (*p* > 0.05)
Ratio of L2SR to m. L2SR	**0.375**	0.0004	21.16	**−62.268 (*p* ≤ 0.05)**	−18.767 (*p* > 0.05)	**−20.919 (*p* ≤ 0.05)**	−3.636 (*p* > 0.05)
Ratio of L3SR to m. of L3SR	**0.401**	0.0002	20.73	**−77.580 (*p* ≤ 0.05)**	−11.781 (*p* > 0.05)	−18.282 (*p* > 0.05)	−0.072 (*p* > 0.05)

AR, annual rainfall; LYAR, last year’s annual rainfall; L2YAR, last 2 years of annual rainfall; SR, season rainfall; LYSR, last season’s rainfall; L2SR, last 2 season’s rainfall; L3SR, last 3 season’s rainfall; m, mean. The values in bold font are significant *p* ≤ 0.05.

Seasonal livestock prices alone were not significant predictors of SWC (*p* > 0.05; Table 3). However, using seasonally adjusted regression, the ratio of L3SR and m. L3SR (RL3SR) and all the livestock price combinations (cents per kilogram carcass weight), were significant predictors of SWC. The combination of rainfall and livestock prices were better predictors of SWC than rainfall or livestock prices alone. The adjusted R square for the price of heavy lamb and RL3SR was highest (0.59), followed by merino lamb and RL3SR (0.588), goat and RL3SR (0.545) and steer RL3SR (0.478), and all were significant (*p* ≤ 0.05).

**Table 3:** Results from regression analysis comparing stock prices, c/kg cwt (Australian dollars) to SWC and the same livestock prices with seasonal rainfall compared to SWC.

Variable compared to SWC	Adjusted R Square	Significance F	Standard Error	Regression coefficient and *(p value)*			
				Winter	Spring	Summer	Autumn
Seasonal stock prices (₵/ kg cwt) compared to the total number of SWC							
Seasonal medium steer price 4-500 kg (c/ kg cwt)	−0.040	0.643	27.314	−0.024 (*p* > 0.05)	−13.918 (*p* > 0.05)	−14.035 (*p* > 0.05)	−1.601 (*p* > 0.05)
Seasonal heavy lamb price 22 + kg (c/ kg cwt)	−0.040	0.401	26.723	−0.046 (*p* > 0.05)	−16.3318 (*p* > 0.05)	−15.469 (*p* > 0.05)	−2.276 (*p* > 0.05)
Seasonal merino lamb price 16-22 kg (c/ kg cwt)	0.049	0.227	26.115	−0.059 (*p* > 0.05)	−17.469 (*p* > 0.05)	−15.523 (*p* > 0.05)	−1.124 (*p* > 0.05)
Seasonal goat price 20.1 + kg (c/ kg cwt)	−0.0005	0.423	26.786	−0.0267 (*p* > 0.05)	−14.005 (*p* > 0.05)	−13.921 (*p* > 0.05)	−1.844 (*p* > 0.05)
Ratio of L3SR to m. L3SR and livestock prices (₵/ kg cwt) compared to total number of SWC							
Ratio L3SR & seasonal medium steer 4-500 kg (c/ kg cwt)	**0.478**	<0.001	19.346	**−87.3420 (*p* ≤ 0.05)**	−12.159 (*p* > 0.05)	−**22.886 (*p* ≤ 0.05)**	−1.664 (*p* > 0.05)
Ratio L3SR & seasonal heavy lamb 22 + kg (c/ kg cwt)	**0.590**	<0.001	17.141	**−92.948 (*p* ≤ 0.05)**	**−16.568 (*p* ≤ 0.05)**	**−24.895 (*p* ≤ 0.05)**	−2.393 (*p* > 0.05)
Ration L3SR & seasonal merino lamb 16-22 kg (c/ kg cwt)	**0.588**	<0.001	17.196	**−86.893 (*p* ≤ 0.05)**	**−16.910 (*p* ≤ 0.05)**	**−23.016 (*p* ≤ 0.05)**	−0.137 (*p* > 0.05)
Ratio L3SR & Goat 20.1 + kg (₵/ kg cwt)	**0.545**	<0.001	18.055	**−88.631 (*p* ≤ 0.05)**	−12.053 (*p* > 0.05)	**−21.329 (*p* ≤ 0.05)**	−1.451 (*p* > 0.05)

(c/kg cwt), cents per kilogram carcass weight (Australian dollar); L3SR, last 3 seasons of rainfall; m, mean. The values in bold font are significant *p* ≤ 0.05.

All three annual rainfall variables were significant predictors of the number of SWC in the Northern country region of Victoria (*p* ≤ 0.05; Table 4). Additionally, LYAR and the mean of AR and LYAR were significant predictors of the SWC for the Central region. The rainfall variables were not predictive of SWC in any other regions of Victoria (*p* > 0.05).

**Table 4:** Results of regression analysis comparing regional annual rainfall and SWC.

Regional annual rainfall compared to SWC	Adjusted R Square	Significance F	Standard Error	Regression coefficient and (*p* value)
Wimmera				
Ratio of AR to m. AR	−0.125	0.970	6.293	0.301, (*p* > 0.05)
Ratio of LYAR to m. AR	−0.029	0.413	6.020	−6.09, (*p* > 0.05)
Ratio of m. AR & LYAR to m. AR	−0.080	0.58	6.167	−5.949, (*p* > 0.05)
Central				
Ratio of AR to m. AR	**0.330**	0.048	23.243	**−105.817, (*p* ≤ 0.05)**
Ratio of LYAR to m. AR	**0.327**	0.049	23.300	**−81.578, (*p* ≤ 0.05)**
Ratio of m. AR & LYAR to m. AR	**0.470**	0.017	20.679	**−120.916, (*p* ≤ 0.05)**
Northern Country				
Ratio of AR to m. AR	**0.271**	0.070	24.017	−58.301, (*p* > 0.05)
Ratio of LYAR to m. AR	**0.384**	0.033	22.069	**−47.422, (*p* ≤ 0.05)**
Ratio of m. AR & LYAR to m. AR	**0.559**	0.008	18.671	**−78.028, (*p* ≤ 0.05)**
Southwest				
Ratio of AR to m. AR	<−0.001	0.348	11.813	−20.654, (*p* > 0.05)
Ratio of LYAR to m. AR	0.043	0.270	11.550	−21.280, (*p* > 0.05)
Ratio of m. AR & LYAR to m. AR	<−0.001	0.348	11.813	−20.654, (*p* > 0.05)
Northeast				
Ratio of AR to m. AR	−0.125	0.990	10.417	0.173, (*p* > 0.05)
Ratio of LYAR to m. AR	0.111	0.184	9.262	−14.545, (*p* > 0.05)
Ratio of m. AR & LYAR to m. AR	−0.012	0.373	9.882	−14.023, (*p* > 0.05)
West South Gippsland				
Ratio of AR to m. AR	−0.098	0.667	15.555	−15.189, (*p* > 0.05)
Ratio of LYAR to m. AR	−0.079	0.574	15.421	19.497, (*p* > 0.05)
Ratio of m. AR & LYAR to m. AR	−0.124	0.940	15.742	2.952, (*p* > 0.05)
East Gippsland				
Ratio of AR to m. AR	−0.165	0.134	4.066	−12.527, (*p* > 0.05)
Ratio of LYAR to m. AR	−0.120	0.858	4.708	1.592, (*p* > 0.05)
Ratio of m. AR & LYAR to m. AR	−0.046	0.458	4.549	−7.875, (*p* > 0.05)
Mallee				
Ratio of AR to m. AR	−0.094	0.645	3.047	−1.443, (*p* > 0.05)
Ratio of LYAR to m. AR	0.241	0.085	2.538	−3.904, (*p* > 0.05)
Ratio of m. AR & LYAR to m. AR	0.114	0.180	2.742	−4.147, (*p* > 0.05)

AR, annual rainfall; LYAR, last year’s annual rainfall; m.AR, mean of annual rainfall; m, mean. The rainfall for each regions has been calculated by calculating the mean of the rainfall from 2 different towns within that region, a per the methodology. The values in bold font are significant *p* ≤ 0.05.

Using seasonally adjusted regression, there was a weak but significant correlation between LSR, L2SR and L3SR and the SWC (*p* ≤ 0.05) in Ballarat and Tatura as seen in Table 5. Winter was the only season that was a significant predictor of SWC in either location.

**Table 5:** Seasonal rainfall in Ballarat and Tatura compared to the number of SWC in those towns.

Variable compared to SWC	Adjusted R Square	Significance F	Standard Error	Regression coefficient, (*p* value)			
				Winter	Spring	Summer	Autumn
Ballarat							
Ratio of SR to m. SR	<0.001	0.422	7.055	−0.250 (*p* > 0.05)	−4.055 (*p* > 0.05)	−3.455 (*p* > 0.05)	−3.686 (*p* > 0.05)
Ratio of LYSR to m. LYSR	0.125	0.073	6.599	**−7.417 (*p* ≤ 0.05)**	−3.598 (*p* > 0.05)	−3.793 (*p* > 0.05)	−2.350 (*p* > 0.05)
Ratio of L2SR to m. L2SR	0.194	0.024	6.329	**−14.945 (*p* ≤ 0.05)**	−4.739 (*p* > 0.05)	−4.710 (*p* > 0.05)	−4.828 (*p* > 0.05)
Ratio of L3SR to m. L3SR	0.177	0.036	6.435	**−17.654 (*p* ≤ 0.05)**	−3.133 (*p* > 0.05)	−4.052 (*p* > 0.05)	−4.605 (*p* > 0.05)
Tatura							
Ratio of SR to m. SR	0.088	0.131	3.309	−2.314 (*p* > 0.05)	−0.3 (*p* > 0.05)	0.06 (*p* > 0.05)	2.7 (*p* > 0.05)
Ratio of LYSR to m. LYSR	0.239	0.010	3.030	**−3.685 (*p* ≤ 0.05)**	−0.3 (*p* > 0.05)	0.244 (*p* > 0.05)	2.774 (*p* > 0.05)
Ratio of L2SR to m. L2SR	**0.176**	0.036	3.181	**−3.895 (*p* ≤ 0.05)**	−0.164 (*p* > 0.05)	0.438 (*p* > 0.05)	3.051 (*p* > 0.05)
Ratio of L3SR to m. L3SR	**0.264**	0.008	3.044	**−6.487 (*p* ≤ 0.05)**	−0.352 (*p* > 0.05)	0.338 (*p* > 0.05)	3.141 (*p* > 0.05)

SR, seasonal rainfall; LYSR, last season’s rainfall; L2SR, last 2 season’s rainfall; L3SR, last 3 season’s rainfall; m, mean. The values in bold font are significant *p* ≤ 0.05.

## 4. Discussion

There were 2,486 SWC affecting key livestock received by the Victorian government between 2011 and 2020. The majority of the farms with SWC had cattle (56%), while 46% had sheep and 4% goats. There were a number of properties that had more than one species of livestock. Based on the data available it was not possible to determine the proportion of all livestock farms that had these key species, however the proportion of all Victorian farm businesses in 2020–2021 that had cattle was 48% and sheep 39% (54). The total number of goat farms in Victoria for this period could not be determined. As farm businesses included those without livestock, it is expected that the proportion of key livestock species would be proportionally higher if only those with livestock were considered. Overall, the proportion of SWC on properties that farmed cattle or sheep seem to reflect broadly the proportion of properties farming those animals in Victoria.

The number of SWC fluctuated markedly between the months and successive years. The highest number of SWC were made in August (late winter). Increased animal welfare incidents in winter have also been reported in the literature (16). Insufficient nutrition may contribute to the late winter peak in SWC, as has been reported by others (3, 5). Pasture growth is minimal in June and July in Victoria (55) and therefore the quantity of pasture is likely to be limiting in August. Pasture that is short also increases worm larval ingestion by livestock, as most worm larvae are on the first 2 cm of pasture (56). In addition, livestock have an increase in energy requirements in the winter due to the cold (57), placing further strain on the pasture supply. Animals in light body condition, with little fat, are also more likely to be negatively impacted by the cold (57). Over September and October the number of new SWC dropped dramatically corresponding with maximal pasture growth in Victoria (55).

There is no other literature that has presented the same type of animal welfare complaint analysis as this study, to the authors’ knowledge. There have been studies that have focused on the human factors that contribute to or are associated with animal welfare incidents on farm (32, 34–36, 58). Other studies report on animal welfare issues identified during routine inspections to determine compliance with the EU animal welfare standards (2, 3, 16), while others have reviewed cases that have been prosecuted (5, 6). Finally, animal welfare incidents have been reviewed with the purpose of identifying key indicators for animal welfare risk (59–61).

The proportion of SWC for each species category varied by up to 35% between years and up to 25% between months. The variation is likely due to a number of factors. In Victoria, sheep, cattle and goats are not uniformly farmed across the state (62). The climate varies considerably across Victoria with marked difference in rainfall (63), temperature (64) and annual pasture growth (55). It is not unusual to have above normal rainfall in one part of the state and below average rainfall in another (65). This is supported by the yearly variation in the number of SWC received for the different species and regions of Victoria. In some years there may be tough seasonal conditions in areas where there are more cattle, but better conditions in other areas where there are more sheep or vice versa. This may result in one species being disproportionately impacted by tough conditions and poor welfare in some years. In addition, seasonal conditions can alter the likelihood of different health and welfare challenges occurring in extensive farming systems. For example, the number of incidents of fly strike (66), exposure (67), footrot (68) and internal parasites can vary considerably between years. Livestock of different species and age groups also vary in their susceptibility to some conditions, for example sheep are more susceptible to internal parasites (69), whereas adult cattle are less so (70). In contrast, cattle are more likely to be impacted by poor pasture growth than sheep because they are physically less able to eat adequate grass when it is short (71). Furthermore, the total number of cattle, sheep or goats in Victoria varies over the years with commodity prices and dry conditions (72). In mixed farming areas with crops and livestock, the number of livestock present can vary considerably from year to year. This might be influenced by factors such as the predicted weather, commodity prices, availability of labor and input costs such as fuel (73). Therefore, the variation in SWC received for each region may in part be due to variation in the number of key livestock present in those regions.

The total number of SWC varied considerably between years. There are a number of confounding factors that may have contributed to the large variation in SWC. Between 2011–2012 and 2012–2013, the large increase in SWC may have been in part due to an increase in use of the Animal Welfare Log, following its inception in 2009. With only 13 SWC recorded in the Animal Welfare log in 2009 and 2010 combined, it would suggest not all complaints were captured in the log in the first few years of its use. In 2020 there were 116 fewer SWC than in 2019, and this was the first year of the Covid-19 pandemic. As Victorians experienced several lockdowns (74), with Melbourne residents locked down for more than 150 days (75), the number of people traveling to regional areas would have been markedly reduced. Potentially this would have decreased the number of opportunities to observe, and therefore report, incidents of poor welfare. The decrease in SWC between 2016 and 2017 could have been in part due to two changes in the recording framework in that year. Firstly the ‘existing investigations’ category was not active prior to 2017, and some repeat complaints for active investigations might not have been identified and wrongly counted as new complaints, artificially increasing the SWC prior to 2017. Secondly, some dairy and beef cattle complaints were recorded under ‘cattle’ in the log until mid-2017, with separate ‘beef’ and ‘dairy cattle’ categories not used until 2016. Potentially therefore, some of the cases included in the analysis before mid-2017, might have included incidents affecting dairy cattle, artificially inflating the SWC prior to 2017. Although the confounding factors listed here may have contributed to the variation in SWC in this data, seasonal variability is likely to have also been a significant issue. As a lack of adequate nutrition is reported to be a common livestock welfare offense (3–5, 7), the variability in pasture quality and quantity with differing seasonal conditions (7, 41) is likely to be relevant in some incidents.

Analyzing the impact of rainfall on the number of SWC is challenging because of the lag in the impact of the reduced rainfall on pasture growth, feed availability and then animal welfare. While the impacts of drought on farmers (76–80) and farming (81, 82) are well documented, a link between rainfall and the number of SWC was less obvious in this study. Unsurprisingly, annual rainfall was much less predictive of SWC than seasonal rainfall. Annual rainfall can be normal despite some seasons in that year having below or above mean rainfall, therefore it is a much less sensitive measure of how the rain can impact pasture growth.

It is unclear why rainfall was predictive of SWC in only two regions in Victoria. The state seasonal rainfall in combination with livestock prices were the best predictors of SWC. Typically, stock prices are lower during dry periods and drought (83) when there is a glut of livestock for sale, as farmers reduce their stocking rates. This can compound the financial challenges for farmers with increases in cost of purchasing feed as well as decreased returns. While rainfall is important for pasture growth, so is temperature (84) and the timing of the rain. For example, a failure to receive autumn rain, prior to the cold weather starting, will dramatically impact on pasture availability through the winter (85). In contrast, high rainfall in the summer may provide unseasonal green pasture, but it may also increase the likelihood of fly strike (86) in sheep and the persistence of worm larvae (87) on pasture contributing to worm burdens. Rainfall, while vital for livestock farming, is only one of the factors that are associated with poor welfare and many complaints were received in years when the rainfall was not limiting.

It was anticipated that rainfall would be a better predictor of SWC at the town level when compared to region or the state, as it would reflect the conditions more accurately, but this was not the case. There was an effect of rainfall on the number of SWC in Ballarat and Tatura, but it was weak. In addition, the state seasonal rainfall was more predictive of SWC than the regional rainfall. This may be because when the state rainfall mean is lower, more areas of the state may be affected by dry conditions and therefore the increase in SWC is more pronounced.

The source of complaints in this study were similar to those reported in previous studies, including the general public (3, 58), the RSPCA (Victoria) (4) and veterinarians (3, 5). As the majority of complaints made to the RSPCA are from the public (88), overall most complaints received by the Victorian government originate from the public either directly or indirectly through the RSPCA (Victoria).

This study has shown that poor welfare of livestock in extensive farming systems continues to be a problem on some farms, as found by others (1–3, 33, 34). It is likely that the complaints received by agencies only represent a portion of the animal welfare issues occurring in the community. Under-reporting and under detection are known to occur in other community based crimes such as gender based violence (89) and child sexual abuse (90). In addition, not all properties and welfare issues are equally likely to be reported. Properties on main roads are subject to a lot of passing traffic and are more likely to be noticed and reported. Whereas farms in more remote areas will be seen by fewer people. Properties that are larger may have the capacity to house animals in paddocks well away from the road, so they are not so visible. Furthermore, some welfare concerns are likely to be more obvious to the public and thus more frequently reported. Examples include sheep with long wool, cattle that are very skinny or animals that are down. Conversely, sheep that are in poor body condition can remain unnoticed (91). As animal welfare has been receiving more community and industry attention (92) it is expected that increasing public awareness will increase the propensity to report welfare incidences in the future (88).

There were some challenges with detailed analysis of the data due to changes in the framework over the recording period, the inability to verify particular details and the size of the data set, covering just 10 years. While it was possible to determine whether the complaints were substantiated or unsubstantiated from the welfare log, the actual details of the specific offenses were recorded in another data base and will be the subject of a future study. The nature of the legislation breach as recorded in the Animal Welfare Log, was based on information provided by the complainant. As this information was not necessarily validated through farm inspection, it was not considered further here.

Despite the limitations of this study, it is the first of its kind. The authors are unaware of any other study that has analyzed livestock welfare complaints over a set period and compared them by month, year, for different species and to rainfall. This study has identified that the number of substantiated welfare complaints involving non-dairy cattle, sheep and goats varies considerably and that rainfall alone cannot account for these variations. Future analysis of welfare investigation details will provide a better understanding of the issues themselves.

## 5. Conclusion

Substantiated complaints about livestock welfare incidents on farms in Victoria continue to be reported. Most SWC are received from the RSPCA (Victoria) and the general public. The number of SWC varies with each month and between years. The proportion of SWC affecting each key livestock species and in each region of Victoria also varies between the years. While there is a relationship between rainfall and the number of SWC, rainfall does not appear to be the only factor involved in incidents of poor livestock welfare. Favorable seasonal conditions are not protective of livestock welfare as incidents occur across the state when rainfall is not limiting. Despite the limitations, the Animal Welfare Log has provided a useful introduction to understanding instances of poor welfare affecting livestock.

## Data availability statement

The datasets presented in this paper are not readily available because of an agreement made with Agriculture Victoria who supplied the data. Therefore, we cannot supply raw data even though they are anonymized. Unfortunately, requests to access the dataset cannot be considered.

## Author contributions

NW, LH, SC, and AF: conceptualization, methodology, and writing review and editing. NW and RS: formal analysis. NW: data curation, writing original draft preparation, and project administration. LH, SC, and AF: supervision. All authors have read and agreed to the published version of the manuscript.

## Conflict of interest

RS is employed by Herd Health Pty Ltd.

The remaining authors declare that the research was conducted in the absence of any commercial or financial relationships that could be construed as a potential conflict of interest.

## Publisher’s note

All claims expressed in this article are solely those of the authors and do not necessarily represent those of their affiliated organizations, or those of the publisher, the editors and the reviewers. Any product that may be evaluated in this article, or claim that may be made by its manufacturer, is not guaranteed or endorsed by the publisher.
