# Supplementary material for: An analysis of substantiated complaints made about incidents of poor livestock welfare, in Victoria, Australia
Source: Front Vet Sci. 2023 Aug 29;10:1242134. doi: 10.3389/fvets.2023.1242134 (PMC10502162; doi:10.3389/fvets.2023.1242134)
Supplement: Supplementary file 1 [file Data_Sheet_1.docx]

**Supplementary Table 1.** Weather stations used to generate mean annual rainfall for each region and the number of years if data that were used to calculate the mean rainfall for each location

| **Region** | **Town 1 -weather station** | **Years of data used for mean rainfall** | **Town 2- weather station** | **Years of data used for mean rainfall** |
| --- | --- | --- | --- | --- |
| Wimmera | Nhil Aerodrome | 2003-2022 | Patyah (Booroopki) | 1998-2022 |
| Central | Bungareee (Kirks reservoir) | 1881-2022 | Yan yean | 1855-2022 |
| Northern Country | Bendigo airport | 1991-2022 | Kotta | 1967-2021 |
| Southwest | Hamilton airport | 1983-2022 | Ararat prison | 1969-2022 |
| Northeast | Wooragee | 1899-2022 | Boho (Honeysuckle Ck U/S Violet town) | 2001-2022 |
| West south Gippsland | Trafalgar, Toseland Road | 1902-2022 | East Sale | 1943-2022 |
| East Gippsland | Bairnsdale (Mitchell river@ Rosehill) | 1942-2022 | Ensay | 1909-2022 |
| Mallee | Mildura airport, gaps exist | 1946-2022 | Charlton (Donald Street) | 1951-2022 (gaps exist) |

**Supplementary Figure 1.** Number of SWC, in each region per year.

**Supplementary Figure 2.** Proportion of SWC, by year, by region.
